# TriplEP-CPP: Algorithm for Predicting the Properties of Peptide Sequences

**DOI:** 10.3390/ijms25136869

**Published:** 2024-06-22

**Authors:** Maria Serebrennikova, Ekaterina Grafskaia, Dmitriy Maltsev, Kseniya Ivanova, Pavel Bashkirov, Fedor Kornilov, Pavel Volynsky, Roman Efremov, Eduard Bocharov, Vassili Lazarev

**Affiliations:** 1 Laboratory of Genetic Engineering, Lopukhin Federal Research and Clinical Center of Physical-Chemical Medicine of Federal Medical Biological Agency, Moscow 119435, Russia; maria.serebrennikova.msu@gmail.com (M.S.); ivanovaka@gks.ru (K.I.); lazarev@rcpcm.org (V.L.); 2 Moscow Center for Advanced Studies 20, Kulakova Str., Moscow 123592, Russia; bashkirovp@mail.ru (P.B.); kornilov.fd@gmail.com (F.K.); r-efremov@yandex.ru (R.E.); edvbon@mail.ru (E.B.); 3 Federal Center of Brain Research and Neurotechnologies, Federal Medical Biological Agency, Moscow 117997, Russia; mal-dima@yandex.ru; 4 Shemyakin-Ovchinnikov Institute of Bioorganic Chemistry, Russian Academy of Sciences, Moscow 117997, Russia; volynski@yandex.ru; 5 Center for Precision Genome Editing and Genetic Technologies for Biomedicine, Pirogov Russian National Research Medical University, Moscow 117997, Russia; 6 Research Institute for Systems Biology and Medicine, Moscow 117246, Russia; 7 Institute of Cytology, Russian Academy of Sciences, St. Petersburg 194064, Russia

**Keywords:** machine learning, functional activity prediction, cell penetrating peptides (CPP), structural-dynamic properties, protein-lipid interaction, intracellular delivery

## Abstract

Advancements in medicine and pharmacology have led to the development of systems that deliver biologically active molecules inside cells, increasing drug concentrations at target sites. This improves effectiveness and duration of action and reduces side effects on healthy tissues. Cell-penetrating peptides (CPPs) show promise in this area. While traditional medicinal chemistry methods have been used to develop CPPs, machine learning techniques can speed up and reduce costs in the search for new peptides. A predictive algorithm based on machine learning models was created to identify novel CPP sequences using molecular descriptors using a combination of algorithms like k-nearest neighbors, gradient boosting, and random forest. Some potential CPPs were found and tested for cytotoxicity and penetrating ability. A new low-toxicity CPP was discovered from the *Rhopilema esculentum* venom proteome through this study.

## 1. Introduction

Cell-penetrating peptides (CPPs) are a class of short peptides, five to fifty amino acid residues in length, capable of forming complexes with macromolecules or microscopic particles to cross various biochemical barriers. This is the largest and best studied class of compounds, capable of autonomously penetrating through cell membranes, while not violating their integrity [1]. They represent a unique group of biologically active compounds that can be used to modulate cellular processes and develop new methods for the treatment and diagnosis of various diseases. In particular, current approaches to deliver macromolecules to the target site, such as viral vectors or membrane disruption, have intrinsic limitations of immunogenicity, high cytotoxicity, and poor efficacy [2], while penetrating peptides are spared from these drawbacks. For this reason, CPPs are widely used in medicine and cosmetology, becoming key elements to enhance drug concentration in hard-to-reach tissues and cells, thus increasing their therapeutic efficacy [3]. In the light of recent scientific discoveries, research in the field of penetrating peptides represents a huge potential for the development of innovative approaches to treat various diseases and improve the quality of life.

The search for new permeation peptides is a complex and multi-step process that requires the combined efforts of researchers from different scientific disciplines. One of the main difficulties is the need to find peptides that not only have a high permeation capacity but are also safe to use. At the same time, the complexity and variety of parameters determining the mode of uptake require experimental testing, for example, using fluorescence microscopy, before a peptide can be definitively identified as a CPP. However, the ability of a peptide to penetrate the lipid membrane can be predicted on the basis of sequence characteristics. Depending on the origin and mechanism of cell membrane traversal, some methods of describing the specific properties of each CPP may be more successful than others [4]. The earliest and simplest method of predicting a peptide’s permeability is to select sequences with a high content of the most common residues, such as arginine or lysine, since most known CPPs have a positive charge. Nevertheless, many experimentally confirmed CPPs transcend these limitations [5]. Other approaches rely on the physicochemical properties of the peptide, not limited to the primary structure, in particular an ML-based framework called BChemRF-CPPred, which is based on descriptors related to the permeability of these structures across cell membranes and the presence of charged polar groups [6].

To speed up and optimize the process, most research to date has focused on using machine learning methods to predict CPPs based on their descriptors, as they can be involved in the de novo design process of CPPs [6,7,8,9,10]. This makes it possible to analyze large amounts of data and identify patterns in the structure and activity of molecules, speeding up the process of selecting potentially active compounds.

Therefore, our goal was to develop an effective predictive algorithm based on machine learning methods that successfully detects novel sequences of safe cell-penetrating peptides. Furthermore, unlike some current studies in this area, we provide experimental validation of our search method. In this sense, in our study, we present a prediction algorithm based on the stacking of k-nearest neighbor, gradient boosting, and random forest models, which were trained using 20 numerical parameters that best describe the difference between CPP and non-CPP classes of the training sample. The ensemble of models optimized by cross-validation produced an algorithm that successfully identified penetrating peptides in a validation sample. It was used to identify potential penetrating peptides on an independent test dataset consisting of some available proteomes and peptidomes from different organisms, which were then chemically synthesized using a solid-phase method and subjected to cytotoxicity and penetration activity evaluation. The study identified a novel low-toxic cell-penetrating peptide CpRE12 (SYQWQQIFYRSLDGSGAKE) from the proteome of *Rhopilema esculentum*. Subsequent NMR analysis revealed that the N-terminus of the peptide forms up to two alpha whorls, whereas the C-terminus has an unordered structure.

## 2. Results

### 2.1. Key Elements of the Algorithm

Penetrating peptides are short chains of amino acids capable of penetrating cell membranes and transporting various molecules or nanoparticles inside cells, thus offering great potential for the development of new drugs. Nevertheless, the discovery of new penetrating peptides is complicated by the diversity of their amino acid structures and physicochemical properties, the complexity and poorly understood mechanisms of membrane penetration and interaction, and the diversity of target cells. We propose to use classification methods to predict the penetrating ability of peptides and select the most promising candidates for further experimental validation. 

**Figure 1:**
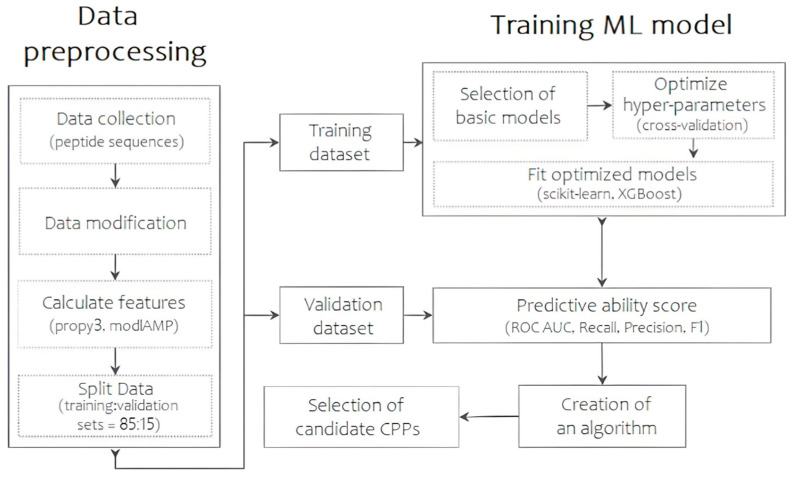
The scheme of the predictive algorithm, reflecting the main steps of data collection and preprocessing, model selection and training, and results evaluation.

Thus, the TriplEP-CPP (Triple Ensemble Prediction of Cell-Penetrating Peptides) algorithm we present is developed based on the stacking of three machine learning models. Figure 1 shows the generalized process of the final prediction model. First, the training sample was assembled using a unified CPP database—CPPsite2.0 [11]. Sequences containing non-natural amino acid residues and synthetic modifications were excluded during data preprocessing. This resulted in 1168 molecules with reliable permeation activity. Similarly, a sample of molecules without permeation activity was obtained from the Swiss-prot database [12]. The main attention was paid to the selection of sequences with high structural diversity, as a result of which the final sample is represented by 1212 amino acid sequences, the percentage of identity of which does not exceed 45%. The training sample is thus represented by 2380 CPP/non-CPP sequences. The distribution of peptide lengths can be found in Figure S1. Each molecule can be represented by a number of numerical features, which become molecular descriptors. Using the propy3 “https://propy3.readthedocs.io/en/latest/index.html (accessed on 21 June 2024)” and modlAMP “https://modlamp.org/ (accessed on 21 June 2024)” packages of the Python programming language, 1134 primary parameters were calculated for each sequence. Descriptors with a correlation coefficient above 0.9 were excluded from further analysis. This resulted in the selection of 774 descriptors that carry a wide range of information about the peptide permeases. As the number of selected descriptors remains redundant, the next step was to obtain an idea of the relative importance of the calculated parameters using the Lasso Tibshirani method “https://scikit-learn.org/stable/modules/generated/sklearn.linear_model.LassoCV.html (accessed on 21 June 2024)”. Based on the results of the analysis, 20 numerical parameters, including descriptors of charge, atomic volume of the molecule, secondary structure, polarization, polarity, solvent accessibility, and instability index, were found to be most influential in determining the differences between the two classes under the conditions set (Table S1). These are the ones that have been the basis for the training of our model. The samples are then divided into training and validation sets in an 85:15 ratio.

At the next step, the most meaningful subsets of parameters of the selected classifiers were tested using the GridSearchCV module “https://scikit-learn.org/stable/modules/generated/sklearn.model_selection.GridSearchCV.html (accessed on 21 June 2024)” from the sklearn class, based on k-nearest neighbors, gradient boosting and random forest methods. The application of tenfold cross-validation allowed us to thoroughly investigate the effect of different parameters on the performance of the models and to maximize their accuracy and predictive power. Details of the list of optimal hyperparameters can be found in Table S2. Thus, for further analysis, the models were trained using hyperparameters aimed at increasing the proportion of correctly predicted CPPs among all possible CPPs. In the final step, stacking, one of the ways to ensemble models, was used to create an overall prediction algorithm by combining the advantages of each classifier to achieve higher prediction precision. The resulting precision value after training and stacking models with adjusted hyperparameters is 0.87. Intermediate results and other metrics are shown in Table S3.

### 2.2. Model Predictive Ability

The results obtained in the predictive ability evaluation were compared with current studies [6,7,8], using a random validation sample of peptides ranging from 5 to 30 aa, 500 CPP/non-CPP sequences each. This comparison of the predictive power of the models allows us to state that the algorithm we have developed is as good as current prediction resources. The results of the comparison of the methods based on accuracy, precision, recall, harmonic mean between precision and recall (F1), and area under the ROC curve (ROC AUC) are shown in Table 1. Of greatest importance to us is the proportion of correctly predicted penetrating peptides among all those predicted as penetrating, as expressed by the precision metrics, and the balance between precision and recall using the f1 metric is also taken into account. Thus, on our proposed datasets, the developed algorithm achieved results comparable to those of other existing models.

### 2.3. Characterization of the Identified Peptides

To conclude, the optimized algorithm was applied to a test dataset represented by 2231528 peptide sequences derived from some available proteomes and peptidomes of different organisms, viz: venom peptidomes of king cobra *Ophiophagus hannah* [13], red ant *Manica rubida* [14], medical leech *Hirudo medicinalis*, hypopharyngeal gland peptidome of honey bee *Apis mellifera* [15], as well as venom proteomes of mygalomorph spiders *Hadronyche infensa* [16] and jellyfish species *Rhopilema esculentum* and *Sanderia malayensis* [17]. Among all the sequences identified by the algorithm as penetrating sequences, seven candidates with some of the best predicted characteristics were randomly selected (Table S4). These were then chemically synthesized by the solid-phase method.

Since the peptides predicted as CPPs are intended to be used as safe intracellular delivery systems, it is necessary to evaluate the degree of their toxicity to mammalian cells. For this purpose, the compounds investigated were added to human HaCaT keratinocyte cells and McCoy mouse fibroblast cells at a final concentration of 100 μM. After daily incubation at 37 °C, the MTT test [18] showed that the percentage of viable cells remained within 82%, indicating that all seven selected predicted CPPs did not affect cell viability (Table S5). 

**Figure 2:**
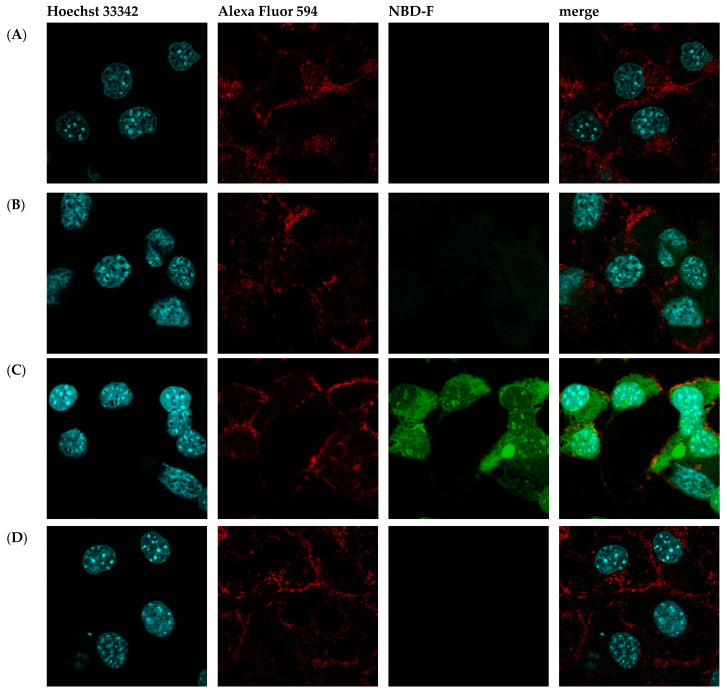

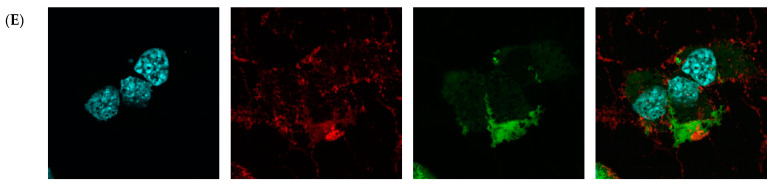
Confocal laser scanning microscopy of internalization of McCoy cells by the predicted cell-penetrating peptide. Mouse fibroblast cells (**A**) without incubation with peptide CpRE12; after incubation with (**B**) NBD-F tag without peptide; (**C**) NBD-F tagged penetratin; (**D**) NBD-F tagged control peptide CpHM15 from the organism *Hirudo medicinalis,* identified by the algorithm as non-penetrating; (**E**) NBD-F tagged predicted peptide CpRE12 from the jellyfish species *Rhopilema esculentum*. Hoechst 33342 intranuclear localization regions are blue; Alexa Fluor 594 cell wall localisation regions are red and peptide-NBD-F localization regions are green. (Scale bar: 106 μm).

Then, fluorescence microscopy was used to evaluate the intrinsic permeability of peptides (Figure 2 and Figure S2). The peptides were labeled with the profluorescent reagent 4-fluoro-7-nitrobenzofurazan (NBD-F), and a tag not conjugated to either compound (Figure 2B and Figure S2B) and the already known permeation peptide Penetratin (Figure 2C and Figure S2C,D) were used as controls. An additional check was made by selecting a peptide from the organism Hirudo medicinalis from the group of peptides identified as non-CPP by the algorithm (Figure 2D and Figure S2E). In accordance with expectations, it showed no penetration activity. As a result, confocal microscopy of McCoy cells incubated with the candidate peptide CpRE12 (SYQWQIFYRSLDGSGAKE) from the jellyfish species Rhopilema esculentum showed that the punctate fluorescence signal was localized both in the cytoplasm and in the nucleus (Figure 2E and Figure S2F–I). Unfortunately, the result for the other six peptides was negative. They show no penetration activity under our experimental conditions (Figure S2J–P).

### 2.4. The Interaction of CpRE12 with Lipid Membranes Does Not Induce Conductive Defects

To investigate the impact of the CpRE12 peptide on the ionic permeability of the membrane, we analyzed its effect on the membrane electrical conductance (Gm) using vertical planar bilayers formed across the aperture in a Teflon cell that separates two KCl aqueous solutions (150 mM KCl, 5 mM Hepes, 1 mM EDTA, pH = 7.0). The ionic current and its noise (fluctuation) flowing through all of the tested membranes (PC:PS:PE 4:3:3; PC:Chol 7:3; PC:PG:PE 4:3:3) remained unaltered after the addition of the peptide to the bulk on either side of the membrane at a concentration equal to 20 μM. The addition of melittin (used as a positive control) to the same membranes led to the formation of conductive defects and subsequent membrane disruption. Thus, CpRE12, when interacting with the membrane surface, only slightly perturbs the lipid bilayers without inducing conductive defects.

### 2.5. CpRE12 Folds into a Flexible Helical Structure and Oligomerized in a Membrane-Mimicking Environment

**Figure 3:**
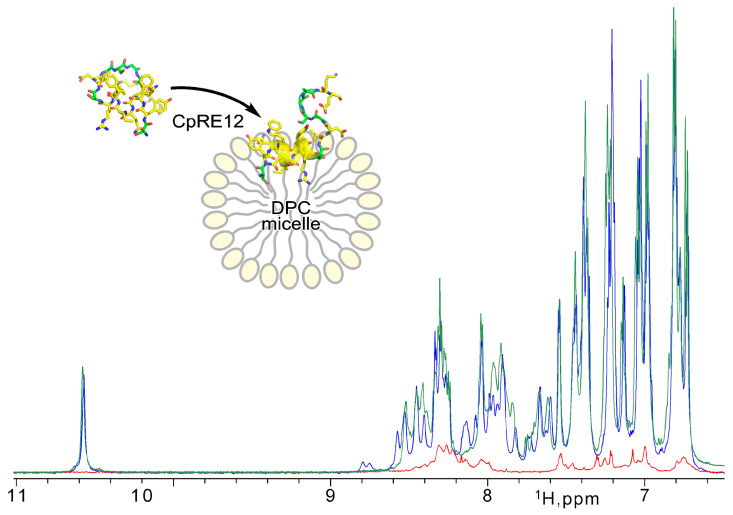
NMR monitoring of CpRE12 structure folding upon its interaction with DPC micelle. Overlaid 1H-NMR spectra acquired for CpRE12 initially dissolved in water buffer (in red) and after addition of micellar suspension at L/P of 60 (in blue) and 200 (in green).

Since the CpRE12 peptide does not have cysteine bridges, it is supposed to be flexible and unstructured in an aqueous solution. Indeed, the ^1^H-NMR spectrum (Figure 3) revealed a strong signal broadening and a small signal dispersion, suggesting a lack of distinct spatial structure and some oligomerization of CpRE12 in water. However, after adding a suspension of DPC micelles to the dissolved peptide sample (Figure 3), the dispersion and intensity of the ^1^H signals increased dramatically, which implies CpRE12 folded into a conformation favoring interaction with the DPC micelle. 

Then, different molar lipid-to-protein ratios (L/P) were tested. The signal assignment and detailed analysis of the NMR spectra acquired at different L/P values (Figure 3, Figures S3 and S4) revealed that CpRE12 undergoes slow-intermediate (on the NMR timescale, in the micro-millisecond range) conformational exchange and dimerization as L/P decreases, with further irreversible oligomerization at low L/P values ultimately. Notably, no precipitation was observed upon CpRE12 oligomerization at L/P of 60 in a micellar environment, and the sample remained transparent. The estimated size of the CpRE12 oligomers surrounded by DPC molecules did not exceed a hundred kDa. 

The NMR spectra of monomeric CpRE12 at high L/P (>200) (Figure S3B) revealed a slow-intermediate (on the NMR timescale) conformational exchange between two states (with occupation ~3:2). Apparently, the N- and C-terminal parts of CpRE12 are involved in the observed conformational exchange (Figures S3 and S4). The latter persists upon CpRE12 dimerization detected upon decreasing L/P, but oligomerization has a significant effect on it, favoring one of the conformations (Figure 3 and Figure S4A). 

**Figure 4:**
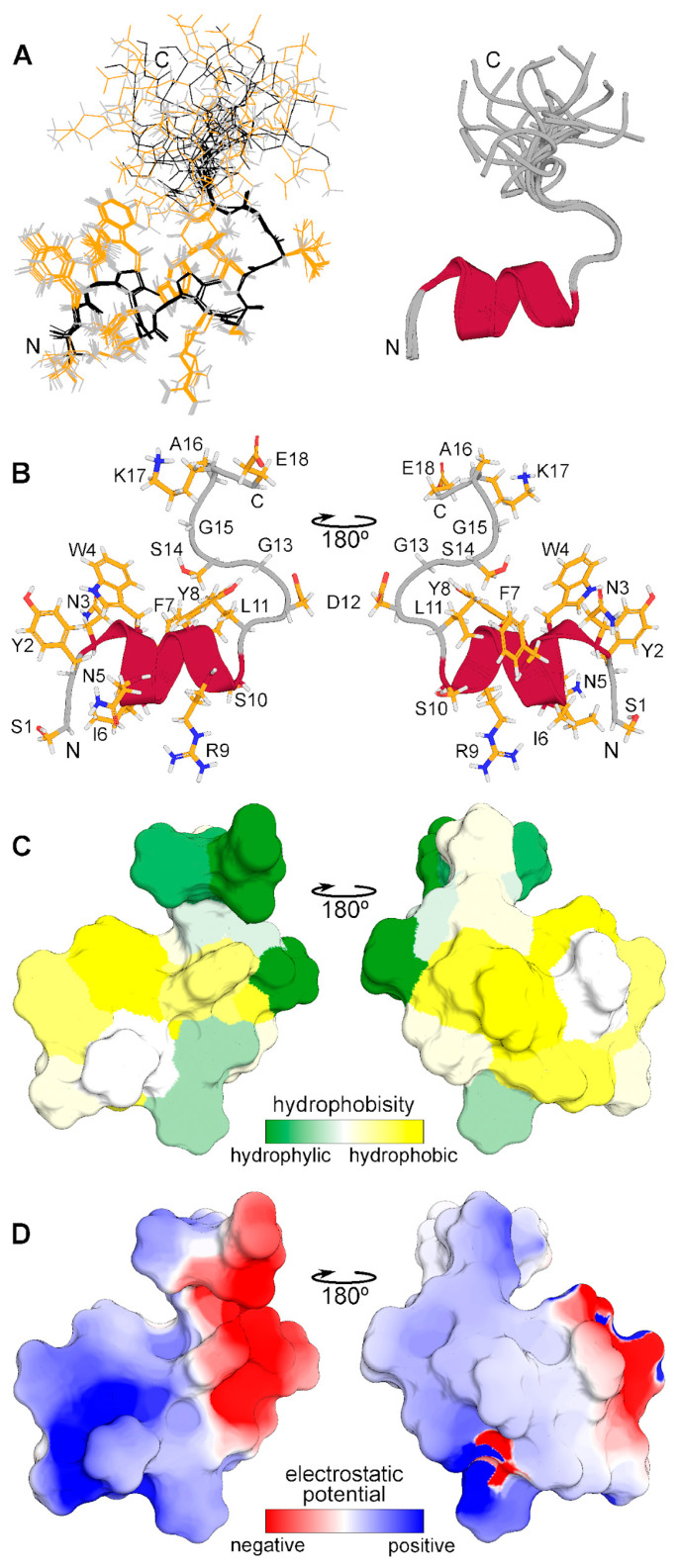
Spatial structure of CpRE12 obtained by NMR analysis in DPC micellar environment. (**A**) Superposition of 12 NMR structures with the lowest target function aligned over the backbone atoms of the folded N-terminal helical part (residues 1–14). Backbone and side chain heavy atom bonds are shown in black and yellow, respectively. Superimposed ribbon diagrams of the NMR-derived structures of are presented on the right. (**B**) Representative NMR-derived structure of CpRE12. (**C**) Molecular hydrophobicity potential (MHP) distribution on the CpRE12 surface. Green is the most hydrophilic (MHP ≤ −3.6), yellow is the most hydrophobic (MHP ≥ 2.1). MHP values are given in logP units, where P is the octanol/water partition coefficient. (**D**) Molecular electrostatic potential (MEP) distribution on the CpRE12 surface. Red is the most negative (MEP ≤ −3 kt/e), blue is the most positive (MEP ≥ 3 kt/e) potential.

The spatial structure of monomeric CpRE12 (major form) was studied by standard NMR methods. According to the NMR structure calculation, the peptide has a relatively stable N-terminal part (residues 1–14) with a short α-helix represented by two turns (residues 3–11), while the C-terminal part is rather flexible (Figure 4). The molecular surface of the folded N-terminal part is amphiphilic with positively charged hydrophilic and large hydrophobic areas, the flexible C-terminal part is hydrophilic and has both positively and negatively charged residues. All resonance assignments and NMR-derived structures have been submitted to the Protein Data Bank (PDB identifier for the CpRE12 peptide is 8R89). 

**Figure 5:**
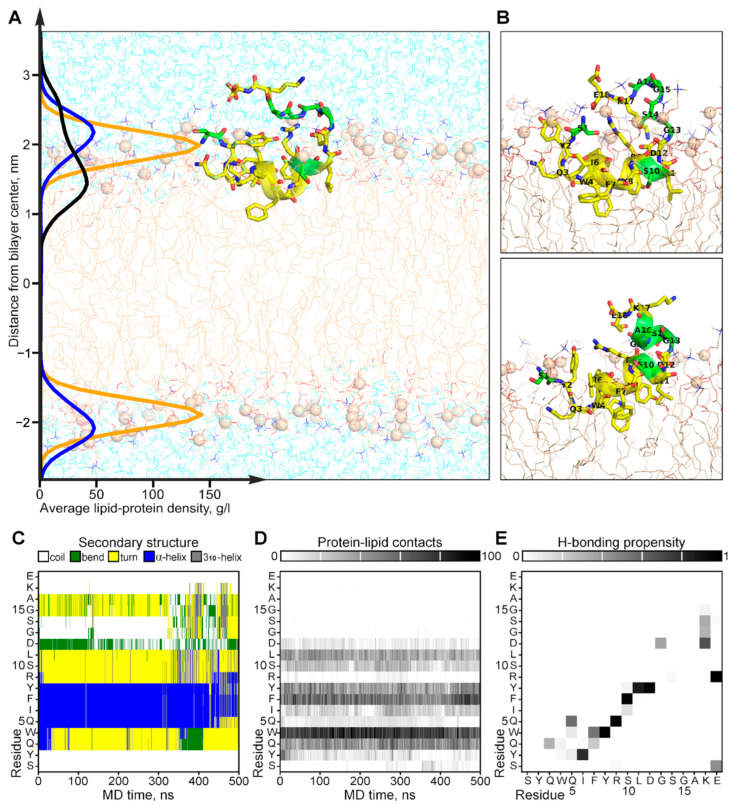
Results of MD simulation of the NMR-derived structure of CpRE12 in POPC bilayer. (**A**) Representative MD snapshot with CpRE12 embedded into hydrated explicit POPC bilayer. The peptide is given in ribbon presentation, glycine, alanine and serine residues are shown in green. Phosphorus atoms of the lipid headgroups are shown by orange spheres. The density distributions of the peptide (in black), phosphorous (in yellow) and choline (in blue) groups of lipids, averaged over MD trajectory, are presented on the left. (**B**) Alternative conformations of CpRE12 observed in MD simulation. (**C**,**D**) Color-coded representation of the MD time evolution of the secondary structure and protein-lipid contacts of CpRE12 embedded into POPC bilayer. The secondary structure elements are shown in blue—α-helix, in gray—3_10_-helix, in yellow—turn, in green—bend, in white—coil. Protein-lipid contacts are color-coded according to the number of direct van der Waals contacts between atoms with 5 Å distance cut-off from white (0 contacts) to black (100 protein-lipid contacts). (**E**) Propensity of H-bond formation between all backbone and side chain atoms of CpRE12, estimated over the MD trajectory.

The experimentally observed conformational exchange and interaction with the membrane of CpRE12 were confirmed and clarified by MD-relaxation of the NMR-derived structure in the explicit POPC lipid bilayer (Figure 5). According to the MD simulation, the folded N-terminal helical part CpRE12 (Figure 5C and Figure S4A), having a distinct pattern of protein-lipid interface formed by hydrophobic and aromatic side chains of residues (Figure 5D), is submerged into the membrane surface under the phosphorous groups of lipids (Figure 5A). The flexible C-terminal part CpRE12 is exposed to water and undergoes conformational exchange with transient folding into a helical turn (residues 13–17) stabilized by local H-bonding in the region 12–15 and the formation of a salt-bridge between the side chains of R9 and E18 (Figure 5C and Figure S5B,C). In addition, a transient long-range H-bond formation is observed between S1 and E18, bringing the N- and C-termini of CpRE12 closer to each other. 

## 3. Discussion

In the world of biologically active molecules, peptides occupy a special place due to their diverse functions and potential applications in medicine. Within this diversity, one interesting class of peptides stands out—cell-penetrating peptides, which have the ability to penetrate cell membranes without disrupting their integrity. Their unique properties offer opportunities for the development of new methods of drug delivery and molecular diagnostics, as well as the creation of tools for the study of cellular processes. Systematic research on CPPs is of great importance for the future development of medicine and biotechnology. Bioinformatics analyses of the proteomes and peptidomes of living organisms actively involve the use of machine learning techniques, which now appear to be a very promising strategy for finding new cell-penetrating peptides. In turn, the combination of biological experiments with computational methods opens up new opportunities to quickly and efficiently process and analyze a large number of factors and data, and to validate preliminary conclusions. Adding machine learning techniques to the search process can identify patterns, improve the quality of predictions, and facilitate subsequent experimentation and development, while reducing costs by weeding out unpromising sequences.

There are many modern computational methods [6,7,8,9,19,20] that can potentially facilitate the search for new cell-penetrating peptides. However, despite significant advances in this field, there are certain limitations that should be considered when using them. One key limitation is the availability of modest data for model training. The number of currently known cell-penetrating peptides is severely limited, making it difficult to generate a training sample and potentially reducing the accuracy and applicability of the predictions. In particular, the sequence-based tool C2Pred [7] is trained on a heterogeneous sample represented by a total of 411 experimentally validated CPPs and 411 known non-CPPs. Another problem is the use of parameters that focus only on the amino acid composition and sequence of the peptide without considering its physicochemical properties. Many algorithms can face the problem of ambiguity in predicting peptide permeability, particularly due to the complexity of biological systems and the poorly understood mechanisms of permeation and interaction with cell membranes. For example, the prediction model KELM-CPPpred [19] uses the following input features: amino acid composition, dipeptide amino acid composition, pseudo amino acid composition, and motif-based hybrid features. It is also important to note that although existing algorithms can provide valuable predictions regarding the potential ability of peptides to penetrate cell membranes, they do not always guarantee high accuracy or reliability without subsequent experimental validation to ensure the necessary validity and applicability of the data obtained. Services such as the two-layer prediction framework MLCPPs [8] or the computational predictor SkipCPP-Pred [9] provide us with valuable theoretical and computational tools for finding or predicting penetrating peptides, but do not ensure validation of the applicability of these approaches in biological systems.

Thus, while existing methods are effective in the context of given conditions, they have various limitations that make it difficult to identify the factors that determine the penetrating ability of peptides. Within the scope of this work, we present an algorithm called Triple Ensemble Prediction of Cell-Penetrating Peptides (TriplEP-CPP) that extends the boundaries of the above limitations. It is a fast and efficient prediction system based on the stacking of three optimized machine learning models: k-nearest neighbors, gradient boosting, and random forest. In its construction process, the following four basic steps were taken: data set creation and partitioning into training and test samples, optimization and training of the selected classifiers, ensembling of the trained models to combine the strengths and improve the performance, class prediction on the test sample to evaluate the predictive ability. For the first step, all currently known penetrating peptides free of non-natural amino acids and modifications were selected, making the sample size larger than some previously published prediction services [7,19]. As the amount of data analyzed increases, accuracy, reliability, and statistical robustness are expected to increase, and the likelihood of overfitting is expected to decrease. By increasing the heterogeneity of the non-CPP sample, we also expect to maintain the representativeness of the data. It is also worth noting that the input data for training the algorithms took into account not only the secondary structure parameters but also some physicochemical properties, in particular descriptors of charge, atomic volume of the molecule, polarization, polarity, instability, and instability index. At the next step, the three selected models were optimized using a tenfold cross-validation to achieve a balance between performance, accuracy and interpretability. In our case, the k-nearest neighbors model finds the k-nearest neighbors for each feature in the training sample and builds on their classes to predict the class of the new feature; gradient boosting is a model training method where each successive model compensates for the errors of previous models; and random forest is an ensemble of decision trees where each tree is built on a different subset of features and objects. The next step is to use stacking to combine the strengths of each of the above algorithms. For example, random forest and gradient boosting can work with an ensemble of trees, which provides robustness to incorrect or inaccurate data. At the final step, after model stacking, the precision of the algorithm on the test sample was 87%, which seems to be a rather encouraging result.

Nevertheless, it is worth considering that experimental validation plays a crucial role in demonstrating the real utility and applicability of the proposed algorithms and approaches. It allows us to identify limitations and potential areas for improvement of the algorithms, as well as providing an opportunity to test how close the theoretical predictions are to real biological processes. Therefore, in our study, seven candidate sequences with a high probability of being CPPs were selected using the TriplEP-CPP algorithm on an independent dataset. Sequences from the proteomes and peptidomes of several organisms were selected for a more comprehensive analysis. As a result, one, two, and three sequences of putative CPPs were selected for the megalomorph spider and the king cobra, 1, for the jellyfish Ropilema 2 and for the medical leech, respectively. These peptides were then chemically synthesized using a solid-phase method.

One of the most important criteria in the search for new penetrating peptides is to ensure the safety of their further use and to assess potential problems and risks. Cytotoxicity assessment allows unsuitable peptides to be screened out at an early stage, thereby narrowing down the choice of peptides and focusing on safer and more effective options. In addition, cytotoxicity testing can also help determine a safe and effective dose of penetrating peptides. Therefore, we incubated human HaCaT keratinocyte and McCoy mouse fibroblast cells with our synthesized compounds at a final concentration of 100 μM for 24 h at 37 °C. The percentage of viable cells after incubation with the peptides was calculated using the MTT assay, and it can be concluded that the candidate peptides do not affect cell viability. The McCoy mouse fibroblast line was the least affected by the added peptides. The low cytotoxicity of the candidate peptides studied allowed us to proceed to the next stage of experimental validation of the algorithm, namely the study of their interaction with cells using confocal microscopy. As mentioned above, cell-penetrating peptides are able to cross the cell membrane and reach internal compartments such as the nucleus or mitochondria. The use of a fluorescent tag makes it possible to observe the penetration of the peptides as well as their distribution and localization within the cells. For this purpose, the synthesized compounds were conjugated with the fluorescent tag NBD-F and incubated with cells at a final concentration of 20 mM for 4 h at 37 °C. Of the 7 candidate sequences with a high probability of being permeabilizing peptides, 1 was confirmed to be indeed permeabilizing from the venom proteome of the jellyfish species *Rhopilema esculentum*. This is the sequence CpRE12 (SYQWQIFYRSLDGSGAKE), which was further analyzed by NMR and MD in a membrane-mimicking environment. 

It was shown experimentally that the CpRE12 peptide, when interacts with zwitterionic detergent micelles, folds partially into a helical conformation and can self-associate. MD simulation in an explicit lipid bilayer detailed a submerging of CpRE12 under the membrane surface and detected possible long-range H-bonding between residues from the middle, N- and C-terminal parts of the peptide due to its flexibility. The revealed structural-dynamic properties of CpRE12 imply that experimentally observed dimerization of the peptide can occur in an antiparallel manner (Figure S6), allowing intermolecular H-bonding between the N- and C-terminal parts of CpRE12. Such a fashion of self-association would provide optimal shielding of the hydrophilic and charged groups of CpRE12 located at the dimer interface, and the exposure of the hydrophobic and aromatic side chains of the residues covered the dimer surface into the lipid environment, facilitating the peptide penetration through the lipid bilayer. Note that in a similar study, the Tat_11_ dimer was able to stabilize the membrane pore over a long period of time, in contrast to the monomer [21]. A related study confirms that transduction becomes more significant with increasing dimer concentrations [22].

To summarize the above, a predictive algorithm has been created to speed up the selection and identification of new cell-penetrating peptides, which in turn will contribute to the development of new therapies and treatments. We have taken care to provide a detailed overview of the implications of the data, machine learning methods, and experimental results that validate the efforts made. This work is a necessary step in the development of peptide penetration technology and the creation of effective tools for their detection. We hope that further research in this direction can lead to breakthroughs in biomedicine and the application of new technologies. 

## 4. Materials and Methods

### 4.1. Sample Preparation

#### 4.1.1. Data Pre-Processing

A set of sequences of molecules with permeation activity and those without was collected using the following databases and services: CPPsite 2.0 [11], Swiss-prot [12]. Confirmed cell-penetrating peptides, capable of safely crossing the cell membrane in complex with various cargoes such as nanoscale particles, small chemical compounds, or DNA fragments, were used to create a training sample. Unfortunately, the number of experimentally proven non-CPPs is rather limited, so the sample we used consisted mainly of random peptides previously generated in Swiss-prot “http://web.expasy.org/docs/swiss-prot_guideline.html (accessed on 21 June 2024)”. Similar approaches to generating a negative control dataset have been used in published studies [7,9].

After merging, duplicates and sequences containing non-natural amino acid residues and additional modifications were removed. For non-CPP compounds, sequences with a high level of similarity were independently removed using the CD-HIT server [23] to increase the heterogeneity of the sample (Sequence identity cut-off = 0.45). As a result, we obtained a dataset containing 2380 sequences ranging from 5 to 61 amino acid residues in length, including 1168 demonstrating permeation activity and 1212 unable to cross the cell membrane independently. The distribution of peptide lengths can be found in Figure S1. The data were processed using Python programming language tools (version 3.11.3).

#### 4.1.2. Descriptor Selection

For the training dataset, 1104 molecular descriptors reflecting their physicochemical properties were computed using packages propy3 “https://pypi.org/project/propy3/ (accessed on 21 June 2024)” and modlAMP “https://modlamp.org/ (accessed on 21 June 2024)”. The number of descriptors was further reduced to 774 by removing highly correlated (R > 0.9) parameters. At the next stage, the most important descriptors for model training were identified using the Lasso Tibshirani or LASSO method, using the LassoCV function from the sklearn.linear_model package “https://scikit-learn.org/stable/modules/linear_model.html (accessed on 21 June 2024)”, which allowed optimizing the predictive ability of the models by reducing the dimensionality of the properties while preserving the maximum amount of information and eliminating overfitting. Thus, 20 parameters were defined, which are further used for model training. They can be divided into seven main groups. Six of them belong to the propy.CTD module, which allows the calculation of descriptors describing general properties of amino acids, the frequencies with which these properties change along the entire length of the protein, and the nature of the distribution of properties along the sequence. The seventh one is represented by a single descriptor defining the instability index and refers to the modlamp.descriptors module. More details on the list of descriptors can be found in Table S1.

### 4.2. Computer Modeling

The predictive algorithm was implemented using the sklearn and xgboost libraries. The total aa sequence data were split into training and delayed samples in the ratio of 85:15. The split was performed using the train_test_split function from the sklearn.model_selection module “https://scikit-learn.org/stable/modules/generated/sklearn.model_selection.train_test_split.html (accessed on 21 June 2024)” with the following parameters: train_size = 0.85, shuffle = True (data shuffling), random_state = 40.

The optimal hyperparameter values are chosen based on the quality metrics on the validation sample, namely accuracy, precision, recall, harmonic mean between recall and precision, and the area under the ROC curve.

The libraries sklearn.neighbors “https://scikit-learn.org/stable/modules/generated/sklearn.neighbors.KNeighborsClassifier.html (accessed on 21 June 2024)”, xgboost “https://xgboost.readthedocs.io/en/stable/ (accessed on 21 June 2024)” and sklearn.ensemble “https://scikit-learn.org/stable/modules/generated/sklearn.ensemble.RandomForestClassifier.html (accessed on 21 June 2024)” were used to build machine learning models. Accordingly, we implemented the k-nearest neighbors method based on the principle of proximity of objects in the feature space, the gradient boosting method based on the idea of sequentially adding weak models and training each next model on the errors of the previous ones, and the random forest method, which is an ensemble of models where, unlike gradient boosting, each tree is built on a random subsample of the training sample and a random subset of features. The search for optimal hyperparameters was implemented using the grid-search method “https://scikit-learn.org/stable/modules/generated/sklearn.model_selection.GridSearchCV.html (accessed on 21 June 2024)” of the sklearn library, applied to the processed dataset using tenfold cross-validation to perform a grid-search of the hyperparameters in the machine learning model. The main evaluation metrics used for cross-validation were precision and accuracy.

For the task at hand, the best result is achieved with the following hyperparameters: for the k-nearest neighbors model {n_neighbors = 18, weights=distance and metric = manhattan}, for the gradient boosting model {n_estimator =250, max_depth = 8, learning_rate = 0.0567 and booster = gbtree}, for the random forest model {n_estimators = 20, max_features = log2 and criterion = gini}. Their combination was implemented by the ensemble method StackingClassifier “https://scikit-learn.org/stable/modules/generated/sklearn.ensemble.StackingClassifier.html (accessed on 21 June 2024)” of the sklearn.ensemble library.

All computational studies were carried out using various modules of the Python programming language. The script and related materials are freely available at the following address “https://github.com/marurser/TriplEP-CPP (accessed on 21 June 2024)”.

### 4.3. Model Verification

Proteomes and peptidomes from various organisms were used to create a sample in which to search for candidate sequences. The peptidome of the king cobra *Ophiophagus hannah* is represented by 578 aa sequences obtained from a study of snake venom gland products [13]. The peptidomes of the ant *Manica rubida* venom [14] and the honeybee *Apis mellifera* hypopharyngeal gland [15] are represented by 4402 and 7501 aa sequences, respectively. The secretion of the medical leech *Hirudo medicinalis* was obtained in the laboratory of genetic engineering of FGBU FNCM FMBA of Russia, for which the peptide was then obtained in the laboratory of bioinformatic methods of combinatorial chemistry and biology of IBH RAS. The resulting peptide includes 1983 aa sequences. The proteome of the mygalomorph spider *Hadronyche infensa* is represented by 89,263 aa sequences obtained from a study of spider venom [16]. The proteome of the jellyfish *Rhopilema esculentum* and *Sanderia malayensis* is obtained from a study of their toxins and consists of 6642 aa sequences [17]. Protein sequences from the proteomes were cut into peptides using the cleavage function of the pyteomics.parser module “https://pyteomics.readthedocs.io/en/latest/api/parser.html (accessed on 21 June 2024)” according to one of 35 preset enzymatic cleavage rules.

As a result, 2,314,988 peptide sequences were selected, ranging in length from 9 to 35 residues, with a diversity of amino acid composition limited to 30%. The features were calculated in the same way as described above. Tags (CPP/non-CPP) were then assigned to each peptide sequence using the predictive model constructed. From the total results, seven peptides were randomly selected and used for more in-depth studies.

### 4.4. Validation and Analysis of Peptides

#### 4.4.1. Cell Lines

McCoy mouse fibroblast cell line (ATCC^®^ CRL-1696™) was grown in a CO_2_ incubator at 37 °C, 5% CO_2_ in DMEM medium supplemented with 10% FBS and gentamicin (10 mkg/mL). Human immortalized keratinocyte line HaCaT cells (ATCC^®^ PCS-200-011™) were grown in CO_2_-incubator at 37 °C, 5% CO_2_ in RPMI medium supplemented with 10% FBS and gentamicin (10 mkg/mL).

#### 4.4.2. Peptide Synthesis by the Solid Phase

The peptides were synthesized by the solid-phase method using the N-9-fluorenylmethyloxycarbonyl (FMOC) strategy on a Liberty Blue automated microwave peptide synthesizer (CEM, Stallings, NC, USA) [24]. NBD-F-labeled peptides were obtained according to the manufacturer’s protocol. Peptides were purified by liquid chromatography with a purity of >95% by an AKTA pure chromatography system (GE Healthcare, Chicago, IL, USA). The MALDI-ToF assays confirmed the sequence and degree of purity with a ULTRAFLEX MALDI-TOF/TOF mass spectrometer (Bruker, Fremont, CA, USA). 

#### 4.4.3. Cytotoxicity

The cytotoxicity of the peptides was assessed using the MTT-test (3-(4,5-dimethylthiazol-2-yl)2,5-diphenyltetrazolium bromide) according to a standard protocol [18]. McCoy, HaCaT cells were seeded in 96-well plates to a density of 5 × 10^3^ cells and 3 × 10^3^ cells in each well and incubated at 37 °C, 5% CO_2_ for 24 h. After 24 h incubation, the cells were washed with Hanks’ balanced salt solution (HBSS). The test peptides were tested at a final concentration of 100 µM in five replicates. Fresh culture medium without added peptides was used as a negative control. Melittin, the main peptide component of bee venom (GIGAVLKVLTTGLPALISWIKRKRQQ), was used as a negative control. After adding 100 µL of the test compound to the cells, the samples were incubated at 37 °C, 5% CO_2_ for 24 h. Next, 10 µL of MTT working solution was added to each well, followed by incubating the cells for 4 h and adding 100 µL of solubilizing buffer to dissolve the formazan crystals. The amount of MTT was measured spectrophotometrically at 570 nm and 690 nm using a Multiskan Ascent microplate photometer (ThermoScientific, Waltham, MA, USA).

Results were processed using Microsoft Office Excel. During the evaluation of cell viability in the first step, the difference between the values obtained at 570 nm and 690 nm was found for each group of measurements. Then, dose-dependent curves were plotted for each cell line. The maximum number of surviving cells was found among the group of measurements incubated without the addition of peptide, the minimum among cells incubated with melittin. The next step was to recalculate the values as a percentage of the positive control and obtain the mean values and standard deviation for each peptide. 

#### 4.4.4. Penetrating Activity

McCoy cells (2 × 10^5^ cells/mL) were cultured overnight on glass confocal dishes (SPL Life Sciences Co., Ltd., Pochon, Kyonggi-do, Republic of Korea). After washing with Hanks’ balanced salt solution (HBSS), cells were incubated with 20 mM NBD-F-labeled peptides or peptide-free Opti-MEM (Gibco) for 4 h at 37 °C. Penetratin (RQIKIWFQNRRMKWKK) was used as a positive control. Nuclei were stained with Hoechst 33342 (Invitrogen, Eugene, OR, USA). Cell membranes were stained with Alexa Fluor 594 (Invitrogen, Eugene, OR, USA). After washing for fixation, cells were treated with 4% formaldehyde for 20 min. Fluorescent peptide localization was examined by a Nikon Ti2-E Inverted Microscope (Nikon Instruments Inc., Tokyo, Japan). Images were processed using the Fiji software (version 2.9.9/1.53t) [25]. 

#### 4.4.5. Ion Conductivity Measurements through Lipids Bilayers

PC:PS:CL (1:1:1) and PG:CL:PE (3:2:5) “black” lipid membranes prepared by “painting” technique were used to analyze membrane permeability change upon interaction with the CpRE12 peptide. Membrane conductivity was measured using a vertical cell with divided volumes filled with buffer (150 mM KCl, 5 mM Hepes, 1 mM EDTA, pH = 7.0). Silver chloride electrodes were placed in separate volumes. A 50-mV potential difference was applied to the electrodes, and the ion current was measured using an Axopatch 200B patch clamp amplifier. The stability of the lipid bilayer was checked by observing the membrane conductivity for 10–15 min before adding the studied peptide to the cell volume. Lipid abbreviations: phosphatidylcholine (PC), phosphatidylserine (PS), phosphatidylglycerol (PG), phosphatidylethanolamine (PE), cardiolipin (CL).

#### 4.4.6. Nuclear Magnetic Resonance (NMR) Spectroscopy

The custom synthesized peptide CpRE12 (SYQWQIFYRSLDGSGAKE) was dissolved in 20 mM NaPi buffer, pH 6.2, with 5% D_2_O (*v*/*v*). Purity and identity of the peptide were confirmed by mass spectroscopy. Then, a micellar suspension consisting of deuterated d38-dodecylphosphocholine (d38-DPC, 98%, CIL) was added to the CpRE12 sample with various lipid-to-peptide ratios (L/P) varied from 60 to 800. NMR experiments were performed on the AVANCE III 600 MHz and 800 MHz spectrometers (Bruker Biospin, Ettlingen, Germany) at 30 °C equipped with pulsed-field gradient triple-resonance cryoprobes. High-resolution NMR spectra ^1^H/^1^H-TOCSY (80 ms mixing time), ^1^H/^1^H-NOESY (50 and 100 ms mixing time) and ^1^H/^13^C-HSQC of 2 mM CpRE12 solubilized at L/P = 200 were acquired for chemical shift assignment and structure calculation. The ^1^H and ^13^C resonance assignment was performed via standard procedure based on the analysis of the spectra with the CARA software (version 1.84) [26].

Spatial structure calculations were performed in the CYANA software package version 3.98.13 using the simulated annealing/molecular dynamics protocol [27]. Torsion angles restraints, stereospecific assignment and interproton distance restraints were obtained based on the J-couplings and ^1^H-^1^H NOE (nuclear Overhauser effect) connectivities. The 3JH^N^H^α^ couplings were determined from the line shape analysis of the cross-peaks in the ^1^H/^1^H-TOCSY spectrum. Upper interproton restraints were estimated from the r^−6^ calibration of the cross-peak intensities in the ^1^H/^1^H-NOESY spectra. A survey of the structural statistics for the final ensemble of the 20 NMR-derived structures of CpRE12 in the micellar environment is provided in Table S6. The NMR chemical shifts and coordinates of CpRE12 were deposited to the Protein Data Bank [PDB] “http://www.rcsb.org/ (accessed on 21 June 2024)” under accession ID code: 8R89. 

#### 4.4.7. Molecular Dynamics Simulation of CpRE12 in Model Membrane 

In order to assess the conformational dynamics and intermolecular interactions of CpRE12 in the explicit lipid bilayer, molecular dynamics (MD) simulations were performed using GROMACS 5.1.4 package [28] and Martini2.2 force-field [29] with TIP3P water model [30] and lipid parameters as described elsewhere [31]. The lipid bilayer consisting of 1-palmitoyl-2-oleoyl-sn-glycero-3-phosphocholine (POPC) was taken for modeling the eukaryotic plasma membrane. MD simulations were carried out in two stages. First, the calculations were accomplished in a coarse-grained (CG) representation in order to identify possible peptide-membrane contacts. This was conducted using parameters recommended for modeling systems of similar composition [32]. 

Initially, a membrane of 200 POPC lipids and 3000 unpolarized water nuclei was generated and equilibrated via 1 μs calculations at a constant temperature of 300 K (v-rescale thermostat) and pressure (1 bar, semi-isotropic circuit, Parrinello-Rahman barostat). Next, the peptide was inserted into the aqueous phase of the system from the equilibrated membrane (gromacs insert-molecules with parameter—replace W utility). When creating the topology of the peptide, to reduce artificial changes in its structure, we used the Elastic Network scheme [33] with a cutoff for generating constraints of 0.7 nm and a force constant of 500 kJ/nm^2^. The constraints, resulting in unstable behavior of the distances between the nuclei derived from the NMR structures, were removed from the set of constraints. The resulting systems were equilibrated by energy minimization followed by 10 ns MD calculations with an integration time step of 10 fs. Next, a 5 μs calculation was performed with an integration time step of 20 fs to study the interaction with the membrane. To collect statistics, the calculations were repeated 10 times. 

To study the peptide-membrane interaction in more detail, the CpRE12 conformation having the most common peptide-membrane interface observed in the resulting set of MD-trajectories was further selected and converted to an all-atom (AA) representation. AA simulations were carried out using the Amber14 force field [34] for protein and the Slipids model [35] for POPC. The calculations were carried out at constant temperature (300K, v-rescale thermostat) and pressure (1 bar, semi-isotropic scheme, Parrinello-Rahman barostat). Van der Waals and short-range electrostatic interactions were evaluated using a cutoff of 1.4 nm. Long-range electrostatic interactions were treated using the Particle Mesh Ewald (PME) method with a grid spacing of 0.14 nm. The peptide was converted to AA presentation with a standard algorithm (backward.py). Refinement of the peptide conformation close to the initial NMR structure was made using additional so-called ExpRst constraints imposed in CG simulations on the distances between CA atoms (i) and on the distances (0.2 nm) between backbone H and O atoms, for which hydrogen bonds (force constant 500 kJ/nm^2^) were observed in the experimental NMR models (ii). The peptide structure was equilibrated via three steps with imposed ExpRst: (i) steepest descent energy minimization via 1000 steps; (ii) 1-ns MD NVT for relaxation of steric clashes appearing after resolution transformation, integration step 1 fs; (iii) 1-ns MD semi-isotropic NPT for relaxation of lipid bilayer in CG and AA representation, integration 1-fs step. Then, adaptation of the experimental structure to the membrane environment was carried out during 20-ns MD with applied ExpRst constraints. Further behavior of CpRE12 in the explicit POPC membrane was studied by 500-ns unconstrained MD-simulation. 

The conformational dynamics of the protein and its van der Waals contacts with lipid and water molecules were analyzed using the GROMACS package utilities. In order to map protein-lipid interactions, the numbers of direct van der Waals contacts between atoms within the 0.5 and 0.6 nm distance cut-offs were estimated in the cases of AA and CG MD, respectively. The internal mobility maps were created from the standard deviation of the distances between CA atoms, calculated for the peptide structures obtained from the set of MD frames with 1-ns timestep. MD simulation data were analyzed and visualized with PYMOL (Schrödinger, LLC, New York, NY, USA).

## 5. Conclusions

Efficient search for new cell-penetrating peptides requires not only a broad knowledge of molecular biology and chemistry but also innovative data analysis methods, including machine learning algorithms and reliable in silico evaluation of peptide-membrane interactions. It is necessary to consider not only the penetrability of peptides but also their safety and potential side effects. This requires careful analysis of protein structure and function, conducting experiments at the cellular level, and assessing the toxicity of new compounds. Machine learning methods used for prediction significantly reduce the multitude of potential cell-penetrating peptides. In this study, we have implemented and tested a new search algorithm with which we were able to detect a previously unknown penetrating peptide with potential use in therapy. However, there remains a need for additional testing to evaluate all possible effects arising from the delivery of different agents to target cells. It is also worth mentioning that stunning new research results and related data are published almost daily. On the one hand, this makes it necessary to periodically update the algorithm with new information, but on the other hand, the work can be extended in the future by integrating additional molecular features. Our next steps are focused on refining the algorithm and obtaining results that can further contribute to approaches for developing new, more effective, and safer therapeutic peptide drugs that can improve the quality of life for people around the world. 

## Figures and Tables

**Table 1:** Evaluation of the TriplEP-CPP algorithm based on stacking of k-nearest neighbors, gradient boosting and random forest models in comparison with existing models: a machine learning (ML)-based framework named BChemRF-CPPred or beyond chemical rules-based framework for CPP prediction; a web-server called C2Pred based on the proposed model; a two-layer prediction framework for machine-learning-based prediction of cell-penetrating peptides named MLCPP.

Algorithm	Accuracy, %	F1, %	Precision, %	Recall, %	ROC AUC, %
TriplEP-CPP	98.1	98.1	97.6	98.6	98.1
BChemRF-CPPred [6]	86.2	84.8	93.4	77.7	93.1
C2Pred [7]	83.3	83.8	80.7	87.2	90.4
MLCPP [8]	92.3	92.4	89.5	95.6	97.8

## Data Availability

The datasets used in this research are available at https://github.com/marurser/TriplEP-CPP/tree/main/Organisms (accessed on 21 June 2024).

## Supplementary Materials

The following supporting information can be downloaded at: https://www.mdpi.com/article/10.3390/ijms25136869/s1.
