# Supplementary material for: TriplEP-CPP: Algorithm for Predicting the Properties of Peptide Sequences
Source: Int J Mol Sci. 2024 Jun 22;25(13):6869. doi: 10.3390/ijms25136869 (PMC11241344; doi:10.3390/ijms25136869)
Supplement: Supplementary file 1 [file ijms-25-06869-s001.zip › ijms-3051709-supplementary.pdf]

Table S1. The descriptors chosen for modeling.

Description	Descriptor
Charge	ChargeD1025<br>ChargeD1075<br>ChargeD1100<br>ChargeD3001<br>ChargeD3100
Normalized van der Waals volume	NormalizedVDWVD1001<br>NormalizedVDWVD2025<br>NormalizedVDWVD2075<br>NormalizedVDWVD2100
Secondary structure	SecondaryStrD2025<br>SecondaryStrD2075<br>SecondaryStrD2100<br>SecondaryStrD3025<br>SecondaryStrD3100
Polarisability	PolarizabilityD1100
Polarity	PolarityD1075<br>PolarityD3025
Solvent accessibility	SolventAccessibilityD3025<br>SolventAccessibilityD3100
Instability index	InstabilityInd

Table S2. The optimal hyperparameters.

Parameter	Value	Set of values
**k-nearest neighbors**		
n_neighbors	18	[16, 22] in increments of 1
metric	manhattan	minkowski, manhattan, euclidean, chebyshev
weights	distance	uniform, distance
algorithm	auto	auto, ball_tree, kd_tree, brute
**gradient boosting**		
n_estimators	250	[50, 300] in increments of 50
learning_rate	0.0567	[0.01, 0.057, 0.103, 0.15]
max_depth	8	[2, 10] in increments of 2
booster	gbtree	gbtree, gblinear, dart
**random forest**		
n_estimators	20	[20, 320] in increments of 25
criterion	gini	gini, entropy
max_features	log2	auto, sqrt, log2

Table S3. The most important metrics for the trained classification models.

Model	Accuracy, %	F1, %	Precision, %	Recall, %	ROC AUC, %
k-nearest neighbors	88.0	87.0	87.80	86.2	93.5
gradient boosting	88.2	87.6	86.6	88.6	94.3
random forest	86.8	85.9	86.1	85.6	93.5

Table S4. The selected peptides.

Peptide	Species	Sequence
CpHI4	*Hadronyche infensa*	CRYFHYRQKKHWQL
CpHM5	*Hirudo medicinalis*	SKAADESER
CpHM6	*Hirudo medicinalis*	AIRNDEELNK
CpHM7	*Hirudo medicinalis*	ALVVDNGSGMCKAGFAGDDAPR
CpOH10	*Ophiophagus hannah*	KTWHMVYPGGYDHTRG
CpRE12	*Rhopilema esculentum*	SYQWQIFYRSLDGSGAKE
CpRE13	*Rhopilema esculentum*	TVANFKTNSAAPPAAEPPR

Table S5. Viability of eukaryotic cells after incubation with predicted peptides. Results are expressed as mean ± standard deviation (n = 5).

Peptide, C=0,1 mM	Percentage of Viable Cells, %	
	HaCaT	McCoy
CpHI4	95.3 ± 3.5	95.5 ± 3.2
CpHM5	93.8 ± 2.4	96.3 ± 3.4
CpHM6	92.2 ± 2.9	95.3 ± 3.4
CpHM7	82.2 ± 2.7	96.8 ± 2.2
CpOH10	94.6 ± 3.3	94.4 ± 3.1
CpRE12	84.4 ± 4.0	87.6 ± 4.6
CpRE13	90.6 ± 3.6	89.7 ± 3.3
penetratin	71.8 ± 3.0	70.9 ± 5.3
control (DMEM)	95.6 ± 3.2	94.7 ± 3.9
melittin	0.1 ± 0.1	0.0

Table S6. NMR analysis.

**Distance and Angle Restraints**		
Total NOE	159	
intraresidual	62	
interresidual	97	
Sequential (|i-j|=1)	62	
Medium range(1<|i-j|≤4)	31	
Long range (|i-j|>4)	4	
$^{3}J_{HNH\alpha}$	9	
Bachbone angle φ	9	
Sidechain angle χ_1	3	
**Total restraints/per residue**	**171/9.5**	
**Statistics of the obtained set of structures**		
Cyana target function	2.62±0.08	
Restraints violations		
Distance (>0.3Å)	2	
Angle (>5°)	1	
RMSD (Å)	#1-12	#13-18
Backbone	0.23±0.08	1.43±0.40
All heavy atoms	0.66±0.10	2.25±0.53
**Ramachandran Plot statistics***		
Most favoured regions	92.9%	
Additional allowed regions	7.1%	
Generously allowed regions	0.0%	
Disallowed regions	0.0%	

*Ramachandran analysis was performed with PROCHECK tool on RCSB validation server (deposit.rcsb.org/validate/)

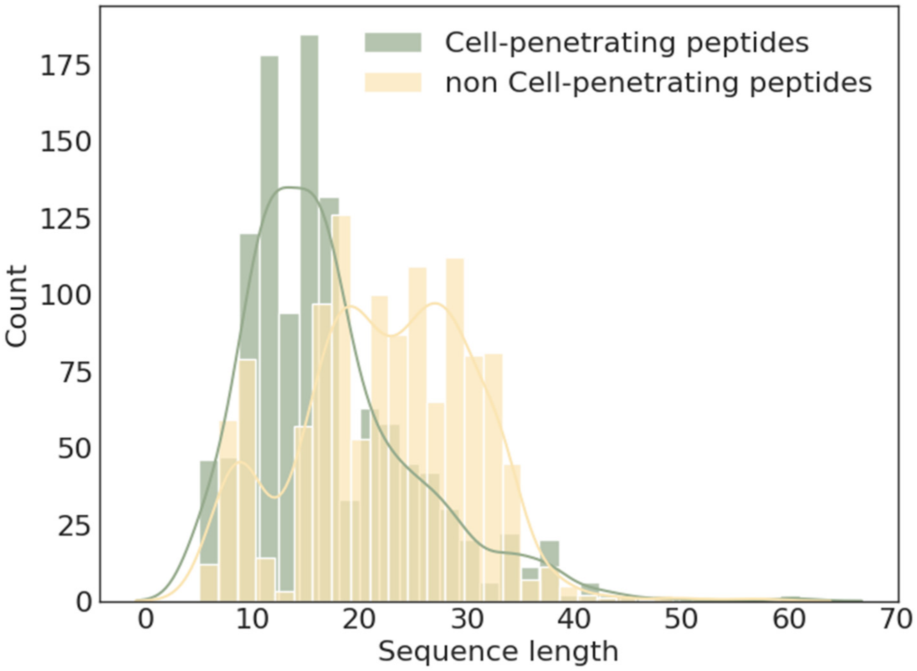

Figure S1. Distribution of peptide lengths in the training sample.

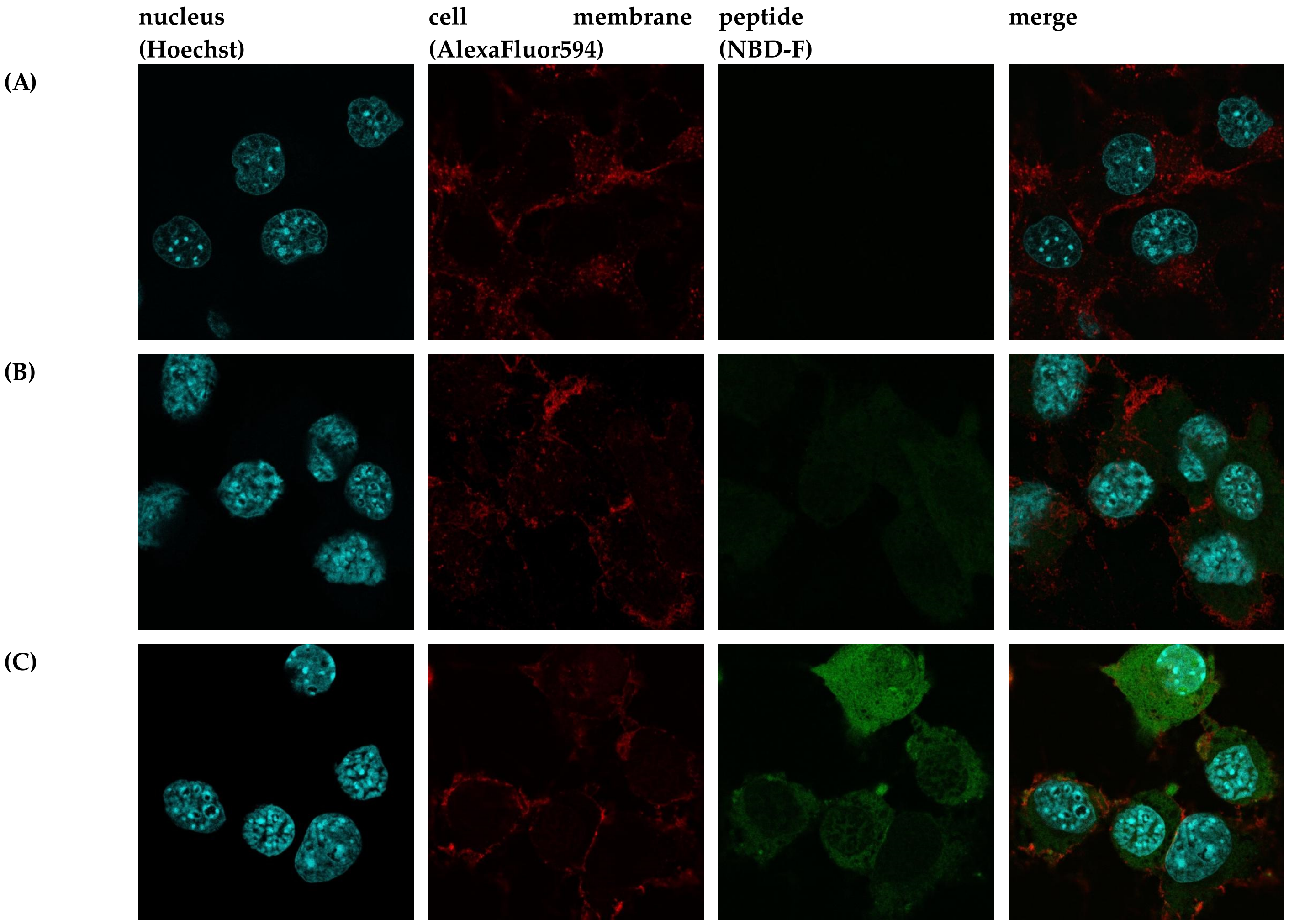

(D)

(E)

(F)

(G)

(H)

(I)

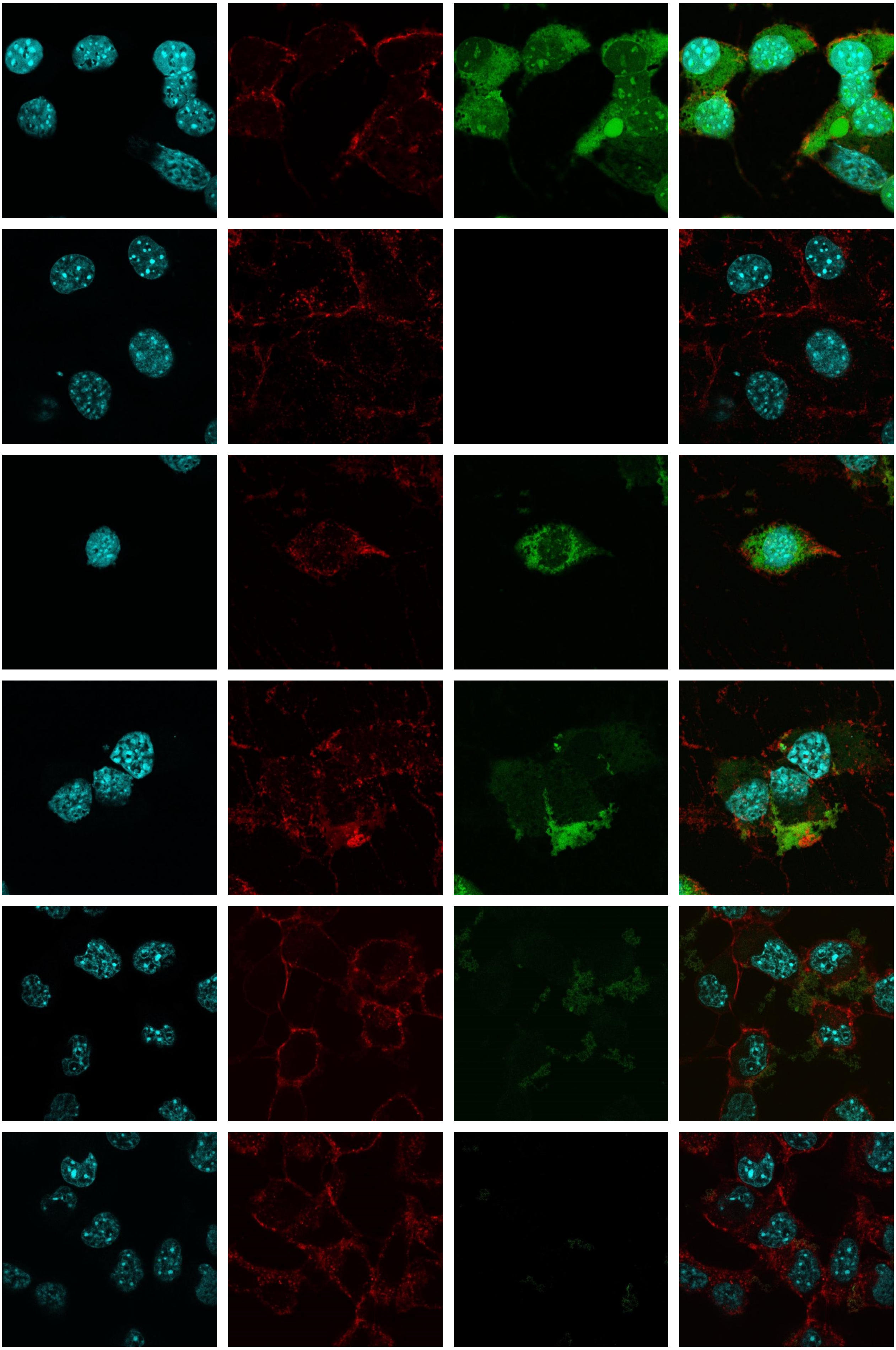

(J)

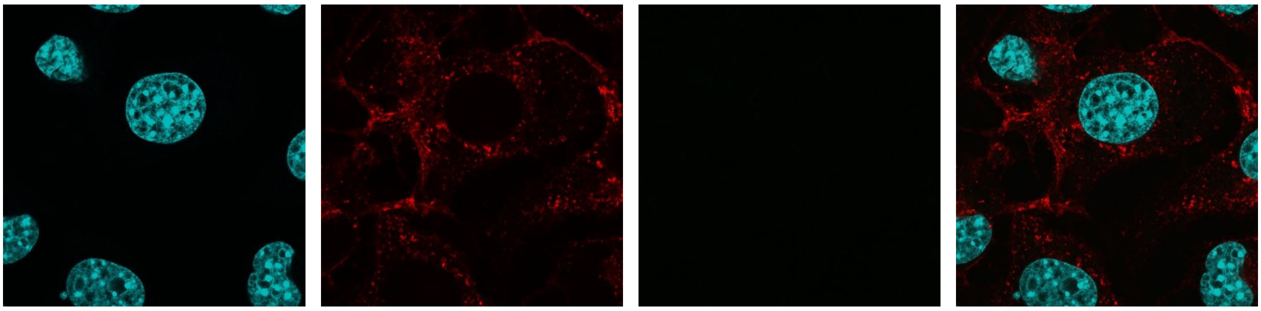

(K)

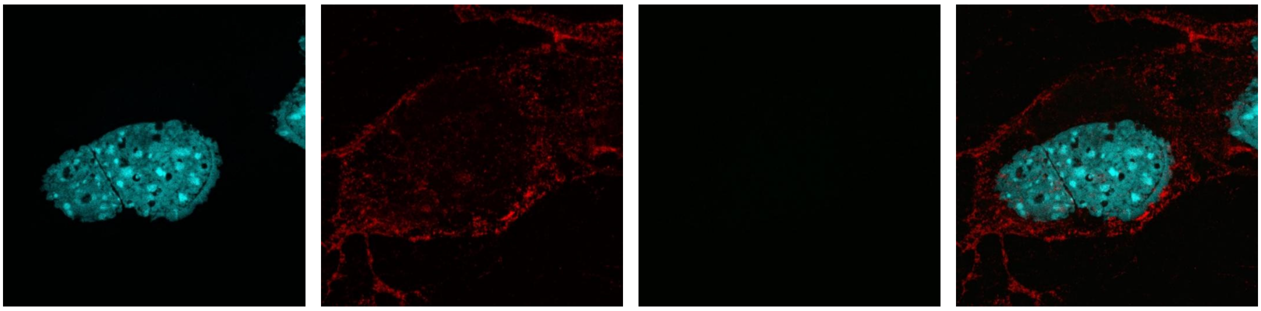

(L)

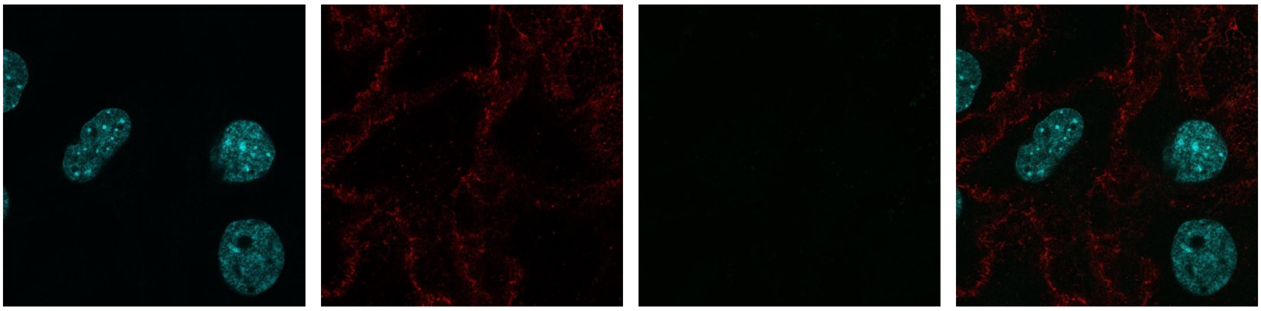

(M)

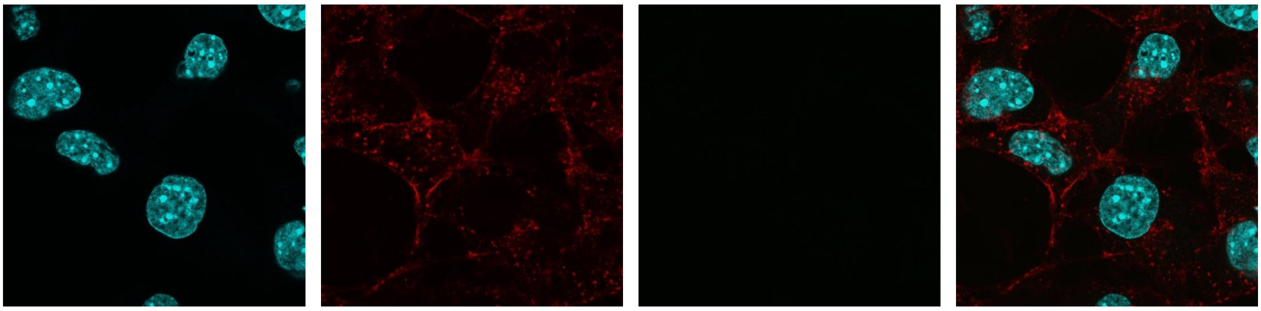

(N)

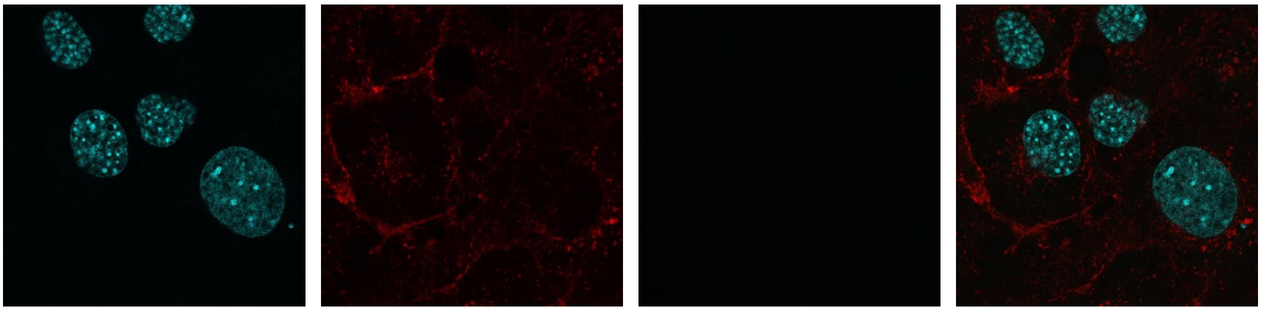

(O)

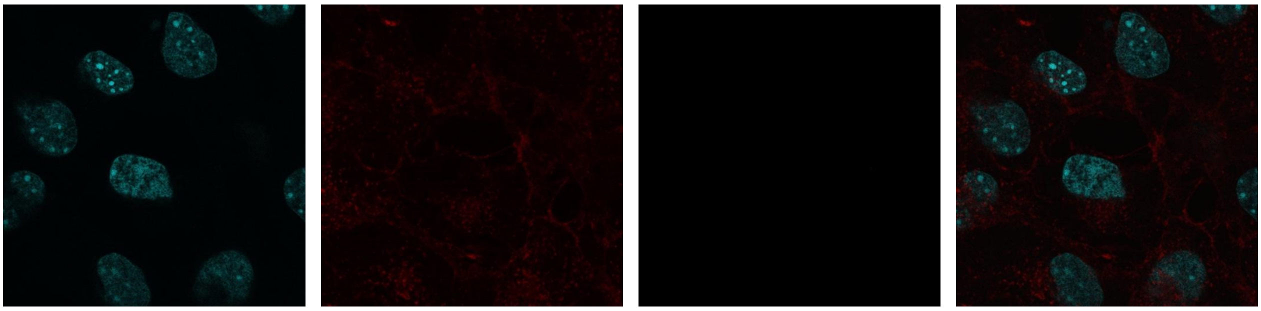

(P)

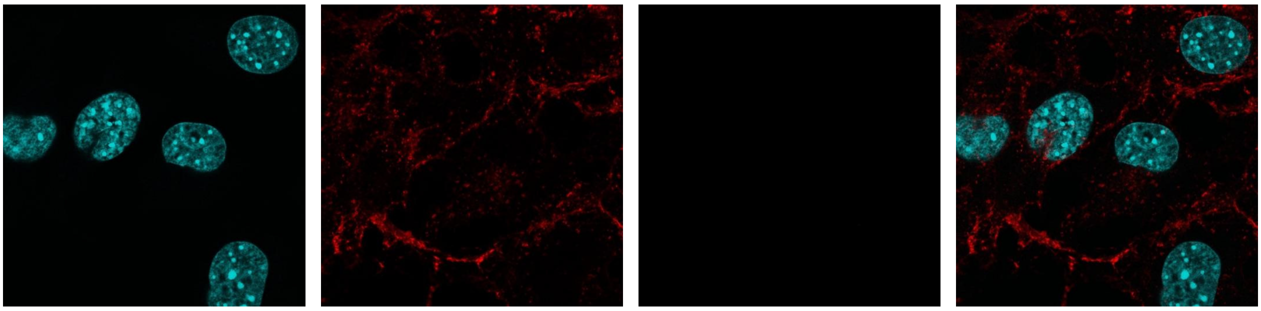

Figure S2. Confocal laser scanning microscopy of McCoy cells internalization by predictive cell-penetrating peptide. Mouse fibroblast cells (**A**) without incubation with peptide; (**B**) after incubation with (**D**) NBD-F tag without peptide; (**C**, **D**) NBD-F tagged penetratin; (**E**) NBD-F tagged control peptide CpHM15 from the organism *Hirudo medicinalis,* identified by the algorithm as non-penetrating; NBD-F tagged (**F-I**) CpRE12 and (**J**, **K**) CpRE13 peptides from *Rhopilema esculentum*, identified by the algorithm as a penetrating; (**L**) NBD-F tagged CpHI4 peptide from *Hadronyche infensa*, identified by the algorithm as a penetrating; NBD-F tagged (**M**) CpHM5, (**N**) CpHM6 and (**O**) CpHM7 peptides from *Hirudo medicinalis*, identified by the algorithm as a penetrating; (**P**) NBD-F CpOH10 tagged peptide from *Ophiophagus hannah*, identified by the algorithm as a penetrating;. Hoechst 33342 intranuclear localisation regions are blue, Alexa Fluor 594 cell wall localisation regions are red and peptide-NBD-F localisation regions are green.

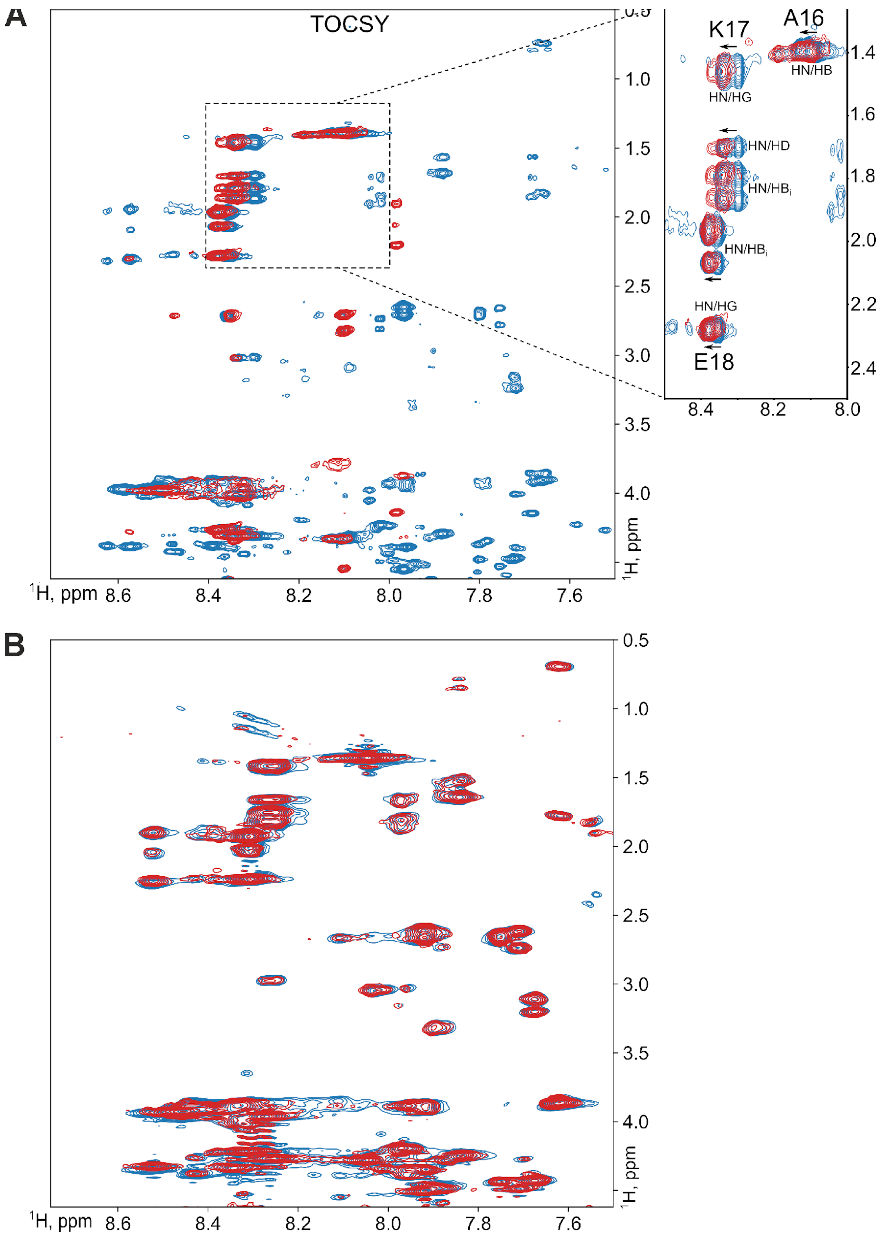

Figure S3. The signal perturbation (splitting, shifting and broadening) observed in NMR spectra due to conformational exchange and oligomerization of CpRE12 solubilized in the micellar suspension at different lipid-to-protein (L/P) ratios. A) Overlaid ^{1}H/^{1}H-TOCSY spectra acquired (on the 800-MHz spectrometer) after addition of the micellar suspension at L/P = 60 to the CpRE12 sample initially dissolved in water buffer (in blue) and repeated 4 days later (in red). In focus of panel (**A**), the cross-peak assignment of A16, K17 and E18 residues. Presumably, CpRE12 undergoes dimerization at L/P = 60 followed by irreversible oligomerization. Indeed, as according to NMR spectra, the increasing of L/P from 60 to 140 does not destroy oligomers previously formed in this sample. The arrows show NMR signal shifting upon CpRE12 oligomerization. Notably, no precipitation was observed upon CpRE12 oligomerization in the micellar environment and the sample remained transparent. Thus, the size of the oligomers surrounded by DPC molecules did not exceed a hundred kDa. (**B**) Overlaid ^{1}H/^{1}H-TOCSY spectra acquired (on the 600 MHz spectrometer) when dissolved CpRE12 solubilized in the micellar suspension at high L/P of 200 (in blue) and 800 (in red). The spectra revealed a slow-intermediate (in NMR timescale) conformational exchange between two CpRE12 states (with occupation ~3:2) exists besides the observed monomer-dimer-oligomer transition. Apparently, N- and C-terminal parts of CpRE12 are involved in the observed conformational exchange (see also **Fig. S4**). As according to the NMR spectra showed on (A), the conformation exchange persists upon CpRE12 dimerization, but oligomerization has a significant effect on it, favoring one of the conformations.

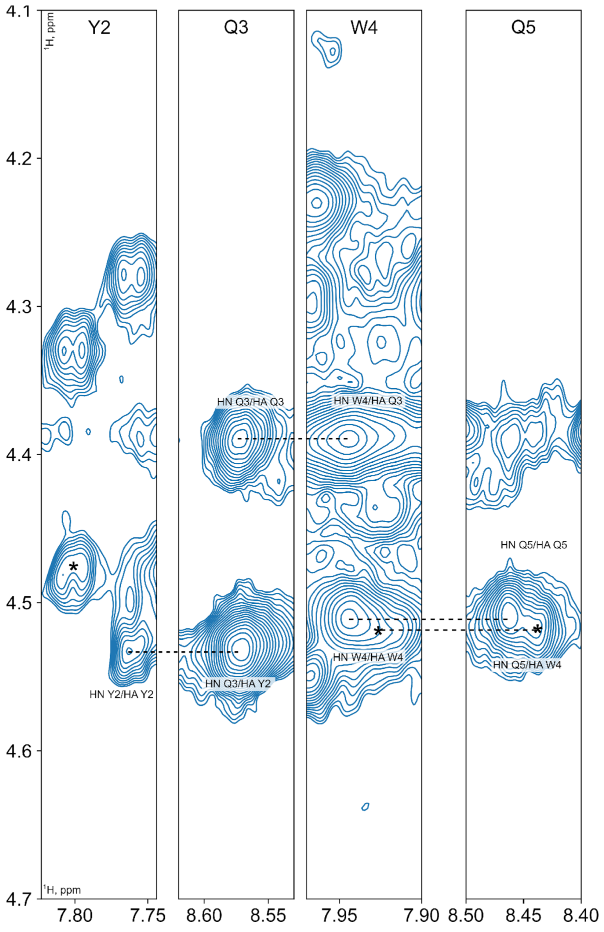

Figure S4. Conformation exchange of CpRE12 revealed by signal doubling in NMR spectra at high L/P values. Representative slices of the ¹H/¹H-NOESY spectrum acquired at L/P = 200 were shown. The cross-peaks of N-terminal residues assigned to minor form of CpRE12 are indicated with asterisks.

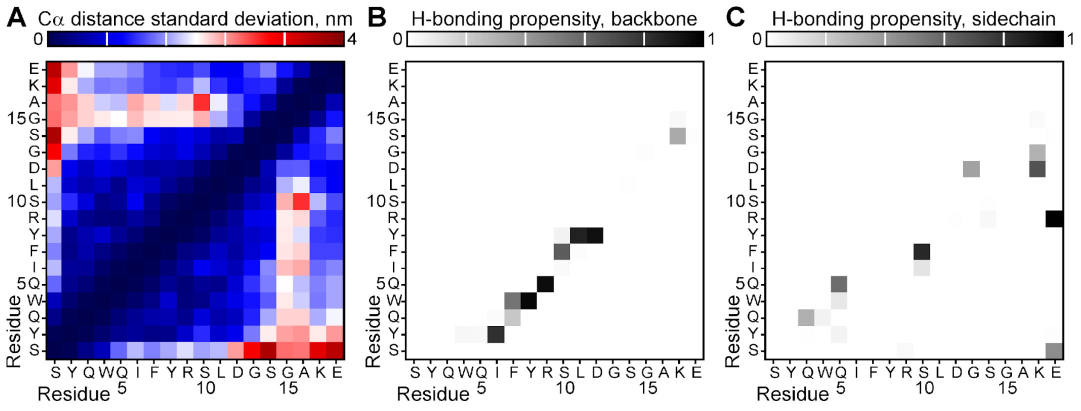

Figure S5. Internal mobility and H-bonding propensity maps obtained from the MD simulation. (**A**) Internal mobility map of CpRE12 derived from the MD simulation. The standard deviation of the distance between the Cα atoms of corresponding residues was calculated overall the frames of MD trajectory. The color-coding scale is shown on the top. (**B**) and (**C**) Propensity of H-bond formation between backbone (only) and side chain atoms of CpRE12, respectively, calculated over the all frames of the MD trajectory.

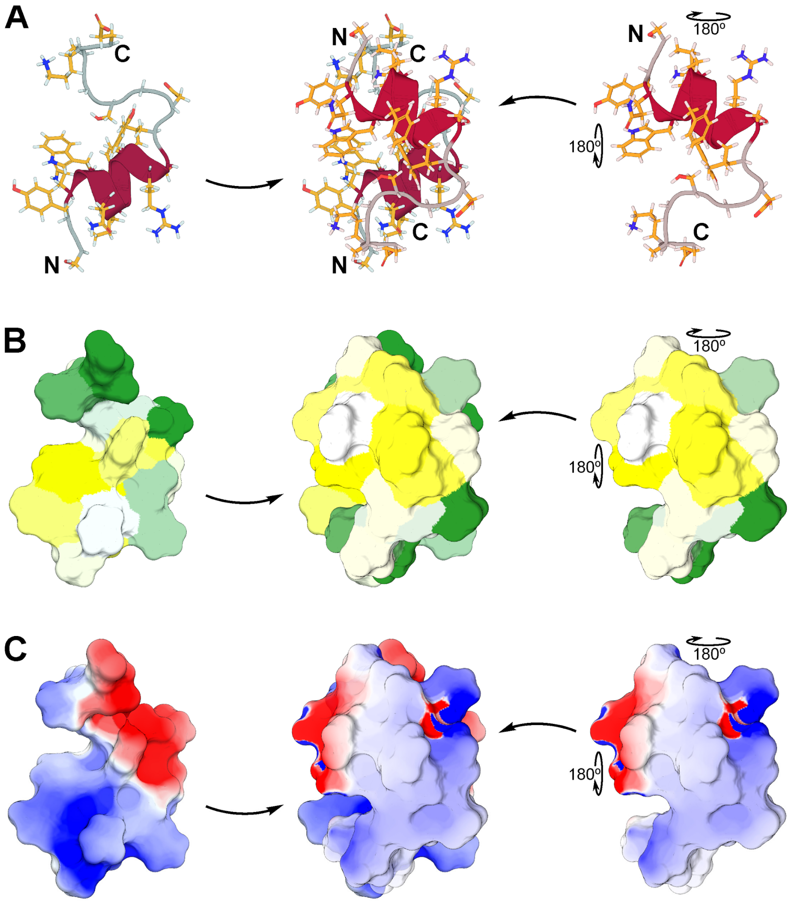

Figure S6. Presumable scheme of an antiparallel dimerization of CpRE12 in lipid environment. (**A**) Two CpRE12 molecules associate in an antiparallel manner, allowing intermolecular H-bonding (e.g. with participation of charged R9, D12, E18, N- and C-terminus) and providing optimal shielding of hydrophilic and charged groups within the dimer interface, while the hydrophobic and aromatic sidechains of Y2, W4, I6, and Y8 are exposed into hydrophobic lipid environment.
